# Comprehensive analysis and effective treatment of plugging in shale gas wells: From composition identification to removal agent optimization

**DOI:** 10.1371/journal.pone.0334495

**Published:** 2025-10-16

**Authors:** Yujia Xiong, Jun Xin, Chao Su, Lei Liang, Qinghua Xiao, Haifeng Ye, Zilai Mei

**Affiliations:** 1 CCDC Geological Exploration and Development Research Institute, Chengdu, China; 2 Sichuan Hengyi Petroleum Technology Service Co. Ltd., Chengdu, China; University of Tehran, IRAN, ISLAMIC REPUBLIC OF

## Abstract

With the extensive exploitation of shale gas fields in southern Sichuan, China, the Weiyuan Area – a key production zone within this region – has experinced a growing gas well plugging problem, which significantly hampers production efficiency. This study presents a comprehensive analysis of plugging problems in this area. Plugging samples were obtained from typically affected gas wells and subjected to a suite of analytical techniques. Results indicated that plugging materials were predominantly inorganic, primarily comprising iron-based impurities and mineral scale deposits, while organic components—present in minor proportions—primarily composed of long-chain alkanes. The formation of these plugs is attributed to downhole corrosion, high-salinity formation water, and complex chemical interactions occurring within the wellbore. In response, specialized plugging removal agents were developed: an organic composite acid-organic solvent system achieved up to 98% dissolution efficiency for iron oxide-dominated plugs; a chelating agent based on CDTA was optimized for iron sulfide-based plugging; and the DTPA-based system exhibited superior dissolution efficiency for barium sulfate/calcium carbonate scale deposits. This research provides a scientific basis for effectively mitigating plugging issues in comparable shale gas fields.

## 1. Introduction

With the continuous growth of global energy demand, shale gas has emerged as a pivotal component in the transition toward increased natural gas production and has become one of the key sources for expanding natural gas output. [1,2]. The shale gas fields in southern Sichuan Province have emerged as key contributors to domestic gas supply, currently undergoing a pivotal stage characterized by large-scale development and enhanced production capacity [3,4]. Within this region, the Weiyuan area stands a significant production zone within the southern Sichuan shale gas field, contributing a substantial portion to the overall gas output [5].However, with the exploitation activities progress in Weiyuan area, the plugging issue has become increasingly severe. Specifically, the annual incidence of plugged wells increased by 37.1% from 2023 to 2024. Futrermore, certain gas wells have experienced repeated plugging events, with recurrence rates reaching up to 20 times, severely disrupting daily production operations.

From a macroscopic standpoint, gas well plugging can be classified into sand plugging [6], scale plugging [7], hydrate plugging [8], and wax plugging [9]. These plugging types are broadly divided organic and inorganic types. Organic plugging predominantly comprises wax, asphaltenes, and materials introduced during drilling and fracturing operations. Inorganic plugging mainly involves the production of formation sand and the deposition of substances such as NaCl, CaCO₃, CaSO₄, BaSO₄, and FeS, wihch are transported by high-salinity formation water. In field applications, chemical plugging-removal methods are predominantly employed, wherein plugging-removal agents are injected into wells to chemically react with and dissolve wellbore plugs, thereby restoring fluid flow. Yang et al. [10] used hydrochloric acid and mud acid to remove wellbore plugging caused by sodium chloride and calcium chloride, achieving a dissolution rate of more than 80%. Tang et al. [11] developed a light-yellow sulfate scale removal agent using various chelating agents to eliminate formation plugging caused by barium sulfate and strontium sulfate precipitation. The dissolution efficiency of this agent for sulfate scale stabilized at 83% after 12 hours. Lightford et al. [12] utilized a water/aromatic hydrocarbon solvent emulsion system to dissolve and remove asphaltenes in the wellbore. In addition, Jones et al. [13] proposed that in case of emulsion plugging encountered in new drilled wells using oil-based or synthetic-based drilling fluids, a micro-emulsion acid system can effectively dissolve the organic and inorganic scales formed.

However, it’s important to note that the types of plugging differs among different gas fields. The chemical plugging-removal method is highly specific, and there is a notable deficiency in systematic and comprehensive research concerning the plugging phenomena and their formation mechanisms within the distinctive geological conditions and production environments of the Weiyuan shale gas field. Consequently, conventional chemical agents achieve a mere 70% success rate in plug removal within this area. Therefore, urgent research is needed to characterize the plugging materials, unravel their formation mechanisms, and develop novel plugging removal agents.

Against this backdrop, this research is dedicated to a comprehensive analysis of the plugging problems in Weiyuan area. It aims to systematically examine the underlying plugging mechanisms and to develop targeted and highly efficient plugging removal agents. The ultimate goal is to establish a specialized plugging removal technology system, thereby enhancing gas well productivity and extraction efficiency. This research is intended to offer valuable references and insights for plugging removal processes in similar shale gas fields [14].

## 2. Materials and methods

### 2.1. Experimental materials

Sample Collecting and Preparing: Plugging samples were sourced from plugged well in the core production zone of Weiyuan area. Priority was given to samples from gas wells that had undergone repeated plugging, as these samples exhibit more complex plugging conditions. These samples were then cleaned with distilled water to remove impurities and then ground into fine powder for homogeneity Water samples were obtained from the same wells for ion composition analysis.

Reagents: All chemicals were used as received. Acetic acid (AcOHl, ≥ 99.5%; Sinopharm) and citric acid (CA,analytical grade,Tianjin Damao) were employed. Sodium dodecyl sulfate (SDS, ≥ 98.5%; Aladdin), polymethacrylic acid (PMAA, ≥ 98%; Aladdin), and diethylenetriaminepentaacetic acid (DTPA, ≥ 99.0%; Sigma-Aldrich) were obtained. Polyaspartic acid (PASP, ≥ 95%; Shandong Xinhua), maleic anhydride-acrylic acid copolymer (MA-AA, 1:3 molar ratio; Tianjin Guangfu), ethylenediaminetriaminepentaacetic acid (EDTA, ≥ 99.0%; Macklin), and 1,2-cyclohexanediaminetetraacetic acid (CDTA, ≥ 99.0%; TCI Chemicals) were also used. An organic acid retarder (SA601; Chengdu Anshide) completed the materials list.

### 2.2. Comprehensive characterization and analysis

Various analytical techniques were employed to examine the composition and structure of the plugging samples. To elucidate the blockage mechanism, detection and analysis were performed on the tubing wall, formation water, and related components [15].

Thermogravimetric ananlysis(TGA): TGA was performed to determine moisture, organic, and inorganic contents. Samples were accurately weighed (m_i_) into a pre-weighed crucible, dried at 105 °C for 2 h, cooled in a desiccator, and reweighed (m_d_) to assess moisture loss. The crucible was then calcined at 600 °C for 4 h in a muffle furnace to constant weight (m_a_). The organic and inorganic contents were calculated using established formulas (1–3).


$$R_{Water}=\frac{m_{i}-m_{d}}{m_{i}}\times 100\%$$ (1)

$$R_{Inorg}=\frac{m_{a}}{m_{i}}\times 100\%$$ (2)

$$R_{org}=\frac{m_{d}-m_{a}}{m_{i}}\times 100\%$$ (3)

Wherein, R_water_ represents the percentage of water content, R_Inorg_ represents the percentage of inorganic content, R_org_ represents the percentage of organic content, m_i_ is the initial mass of the sample; m_d_ is the mass after low-temperature drying at105°C and drying; m_a_ is the mass of ash after high-temperature combustion at 600°C.

Scanning Electron Microscope(SEM) and Energy-Dispersive X-ray Spectroscopy(EDS) Analysis: Morphological characterization of plugging materials and tubing wall was conducted using a SEM (Zeiss EVO MA15) operated at 15 kV with a working distance of 8–10 mm. Elemental composition of plugging materials was then analyzed using EDS(INCA X-act),which was conducted in spot mode with a resolution of 133 eV to semi-quantitatively analyze elemental compositions over a 0–20 keV range.

X-ray Diffraction (XRD) Analysis: X-ray Diffraction was introduced to analyse the relative content of inorganic components..It was conducted on the extracted scale sample (Cu target, 5°–90° scan), using a Thermo Fisher EQUINOX 100 diffractometer.

Fourier-transform Infrared Spectroscopy (FT-IR) Analysis:FT-IR was employed to characterize the organic fraction within plugging samples. The samples were dried and then ground with potassium bromide. The mixture was pressed into a pellet for measurement using an FT – IR spectrometer (Bruker Vertex 80). Scans were carried out in the wavenumber range of 4000 cm^-1^–400 cm^-1^ with a resolution of 1 cm^-1^.

Gas Chromatography-Mass Spectrometry (GC-MS) Analysis: GC-MS was used to assess organic components and content within plugging samples. Chromatographic conditions: DB – 5MS column, 40 °C (4 min), 10 °C/min to 300 °C (30 min), 280 °C vaporization, 250 °C transmission line, He carrier gas (1.0 mL/min), no – split, 1 μL injection. Mass spectrometric conditions: EI source (70 eV), 200 °C ion source, Scan mode (35–500 u), 3 – min solvent delay. Use dichloromethane as syringe-washing solution.

Ion Chromatography (IC) Analysis: Chemical composition of water samples was conducted using (IC with suppressed conductivity detection. Anion and cation concentrations were quantified against NIST-traceable standards, with a detection limit of 0.1 mg/L.

Scaling propensity was assessed via thermodynamic modeling using SI and SAI [16–18]. These indices were calculated based on measured water chemistry parameters (pH, temperature, TDS), alongside ion activity coefficients derived from the Debye-Hückel approximation. The calculation formula is shown in Equation (4)-(5).


SI=pH−K−pCa−pAlK (4)

SAI=2×(K+pCa+pAlK)−pH (5)

Where pH represents pH value of collected water sample, K represents correction factor, pCa represents negative logarithm of Ca^2+^ concentration, dimensionless, pAlK represents negative logarithm of alkalinity, dimensionless.

### 2.3. Dissolution test and corrosion experiment

Preparation of Plugging Removal Agents: Plugging removal agents were prepared using a specific formula: 10% main agent (chosen from 2.1), 4% chelating agent, 2% flow-back aid, 2% corrosion inhibitor, 1% iron-ion stabilizer,ect.

Dissolution Tests: Pulverized plugging sample (m₁) was added to 50 mL of the plugging removal agent in a reaction vessel. The mixture was then heated at 80 °C for 2h in a water bath. After cooling, the residue was filtered and washed,which was then dried at 120 °C until a constant weight was achieved(m_2)_. The dissolution rate was calculated as shown in Equation (6).


$$R=\frac{m_{1}-m_{2}}{m_{1}}\times 100\%$$ (6)

Where R represents dissolution rate, %; m_1_ represents the mass of the initial plugging mass, g, m_2_ represents the remaining mass of plugging after reaction, g.

Corrosion Experiment: Considering that acidic plugging removal agents may cause corrosion to surface equipment and underground pipes at high temperatures, corrosion rate tests were conducted on N80 steel samples under simulated formation conditions. The N80 steel samples were first cleaned with anhydrous ethanol, dried, weighed, and their surface areas were calculated. They were then placed in a reactor with an acid volume of 20 mL/cm^2^. The reactor was heated to experimental temperature at a rate of 3°C/min, maintained at a pressure of 12 MPa, and then increased to 16 MPa. The mixture was stirred at 100 r/min for 4 hours. After cooling, the samples were cleaned, dried, and re-weighed. The corrosion rate ($\nu_{c}$) was calculated as shown in Equation (7):


$$v_{c}=\frac{w_{1}-w_{2}}{S\times t}\times 10^{6}$$ (7)

Where $\nu_{c}$ represents the corrosion rate, g/(m^2^·h), $w_{1}$ represents the mass of the N80 specimen before the reaction, g, $w_{2}$ is the mass of the N80 specimen after the reaction, g, S represents the surface area of the N80 specimen, in square millimeters, t represents reaction time, h.

To further investigate the impact of wellbore temperature and pressure changes on the corrosive behavior of the plugging removal agent, three typical temperature-pressure combinations were selected as static experimental points: low-temperature/low-pressure (80°C/12MPa), medium-temperature/medium-pressure (100°C/14MPa), and high-temperature/high-pressure (120°C/16MPa). The corrosion rates of individual plugging removal agent under these three conditions were tested following the above experimental procedure. Subsequently, stepwise corrosion experiments were conducted, where the experimental temperature was gradually increased from 80°C to 120°C with a total reaction time controlled at 4h. The final corrosion rate was calculated according to Equation 7 to analyze the cumulative effect of temperature changes.

## 3. Results and discussion

### 3.1. Plugging characteristics

**Fig 1:**
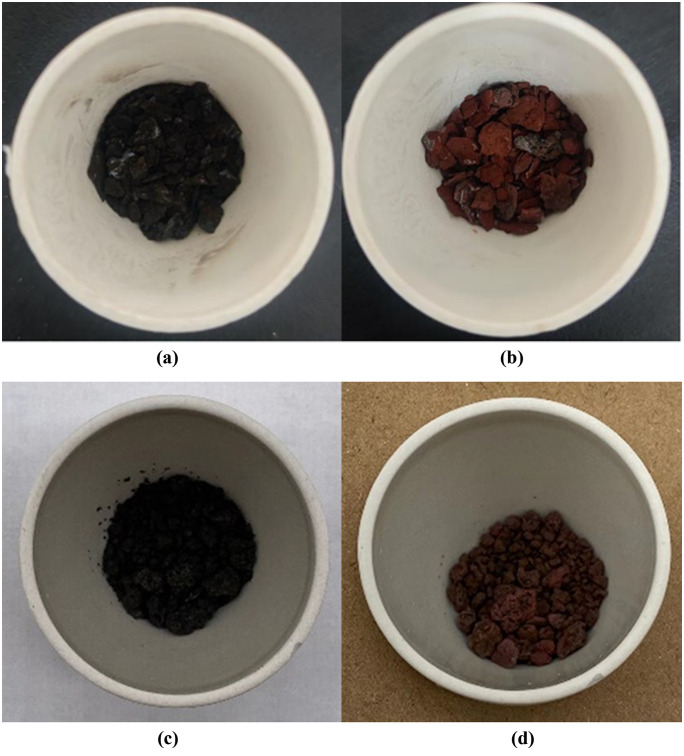
Plugging samples before and after calcination at 600°C. ((a) W-1 before calcination, (b) W-1 after calcination,(c) W-9 before calcination,(d) W-9 after calcination.

**Fig 2:**
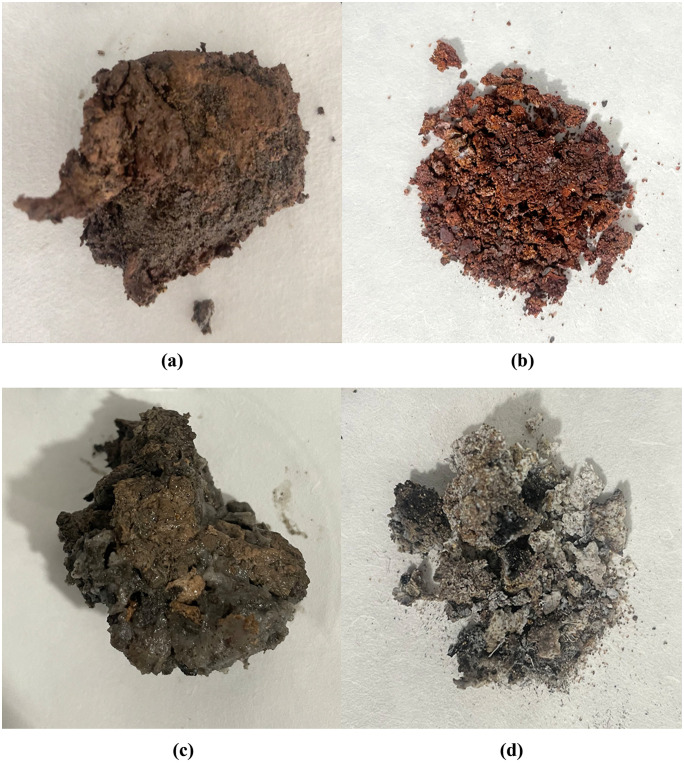
Plugging samples before and after burning calcination at 600°C. ((a) W-9 before calcination, (b) W-9 after calcination,(c) W-10 before calcination,(d) W-10 after calcination).

Quantitative analysis through thermogravimetric experiments determined the relative contents of components within plugging samples. As shown in Table 1, the plugging samples were primarily composed of inorganic constituents, averaging 63.83%,whereas the organic fraction was comparatively low, averaging 17.81%. Notably, only a small portion of plugging samples exhibited a relatively high content of organic matter, which were originally in a colloidal form with wet mud adhesion. As illustrated in Fig 1, the majority of the plugging samples turned reddish-brown after calcination, indicating the existence of iron, while their morphology remained largely unchanged after calcination. Conversely, a small portion of gas wells (taking W-9 and W-10 as examples, as shown in Fig 2) contained plugging material mainly composed of organic matter, with only minimal ash residues remaining after calcination.

**Table 1:** Thermogravimetric Experiment result.

Well number	Weight/g			Content/%		
	Initial	Calcined at 105°C	Calcined at 600°C	Water	Inorganic	Organic
W-1	5.0295	4.4581	4.1723	11.36	82.96	5.68
W-2	6.1345	5.3471	4.6781	12.84	76.26	10.91
W-3	6.6394	5.4120	4.7487	18.49	71.52	9.99
W-4	5.3485	4.6741	4.2731	12.61	79.89	7.50
W-5	6.1433	5.6640	4.9551	7.80	80.66	11.54
W-6	6.3325	5.7940	5.0450	8.50	79.67	11.83
W-7	6.1469	5.6424	5.1560	8.21	83.88	7.91
W-8	6.3260	4.8623	3.2950	23.14	52.09	24.78
W-9	7.3889	5.0832	1.9104	31.20	25.85	42.94
W-10	15.4080	7.7842	0.8473	49.48	5.50	45.02

X-ray Diffraction determined the quantitative inorganic composition of the plugging materials (Table 2), The samples from most wells were predominantly composed of Fe_2_O_3,_ Fe_3_O_4_, and FeCO_3_, with minor amounts of FeS detected. Additionally, certain gas wells contained BaSO_4_ and CaCO_3_; notably, Well W-8 exhibited a relatively high BaSO_4_ content (28.5%), wihle Well W-10 showed a TiO2 content of 26.4%. The compositional analysis of the plugging materials indicated that CaCO_3_ and BaSO_4_ were identified as common scaling products, while Fe_3_O_4_ and Fe_2_O_3_ and FeS were characteristic downhole corrosion products. Additionally, TiO₂, a primary component of fracturing fluid crosslinkers, was also detected.

**Table 2:** XRD Test Results.

Well Number	Fe_2_O_3_/%	Fe_3_O_4_/%	FeCO_3_/%	FeS/%	BaSO_4_/%	CaCO_3_/%	TiO_2_	other/%
W-1	48.6	51.4	0	0	0	0	0	0
W-2	56.4	25.4	4.9	6.7	0	0	0	6.6
W-3	20.8	11.2	62.1	0.8	0	0	0	5.1
W-4	8.1	22.9	67.1	0	0	0	0	1.9
W-5	7.8	61	28.9	1.4	0	0	0	0.9
W-6	5.5	55.6	37.2	0.9	0	0	0	0.8
W-7	9.5	61.3	26.9	1.1	0	0	0	1.2
W-8	10.7	9.1	0	7.7	43.9	28.5	0	0.1
W-9	35.1	48.9	0	3.8	1.5	10.7	0	0
W-10	50.8	12.6	0	10.2	0	0	26.4	0

**Fig 3:**
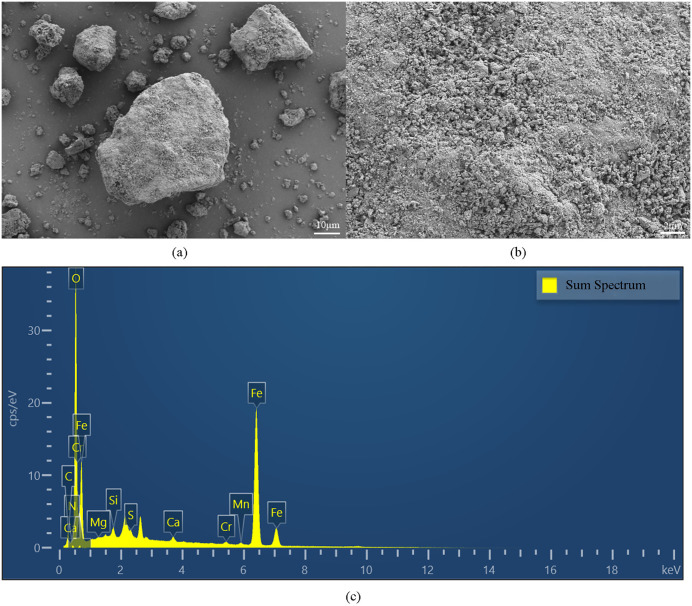
SEM and EDS test of W-1 ((a) SEM micrograph at 10μm scale, (b)SEM micrograph at 2μm scale; (c) EDS test result).

**Fig 4:**
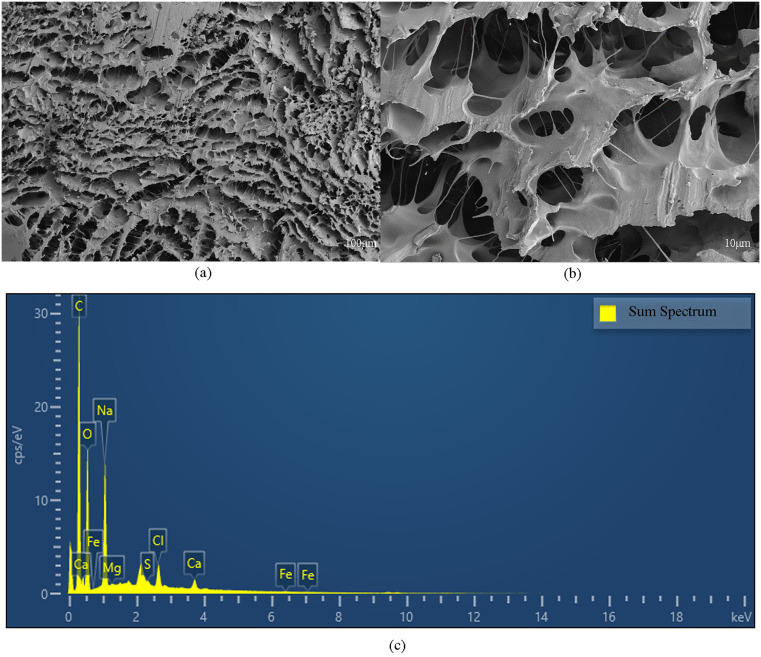
SEM and EDS test of W-9 ((a) SEM micrograph at 100μm scale, (b) SEM micrograph at 10μm scale, (c) EDS test result).

SEM test revealed distinct morphological differences among the plugging samples. As shown in Fig 3, inorganic-dominated plugging sample represented by Well W-1 exhibited a loose, granular to blocky appearance with no visible cementation. In contrast, the material from Well W-9 displayed a filamentous polymeric structure, indicative of distinct organic characteristics (Fig 4). EDS test from W-1 demonstrated that the primary elements was O, Fe, and C, which was consistent with the results obtained from XRD analysis.

**Fig 5:**
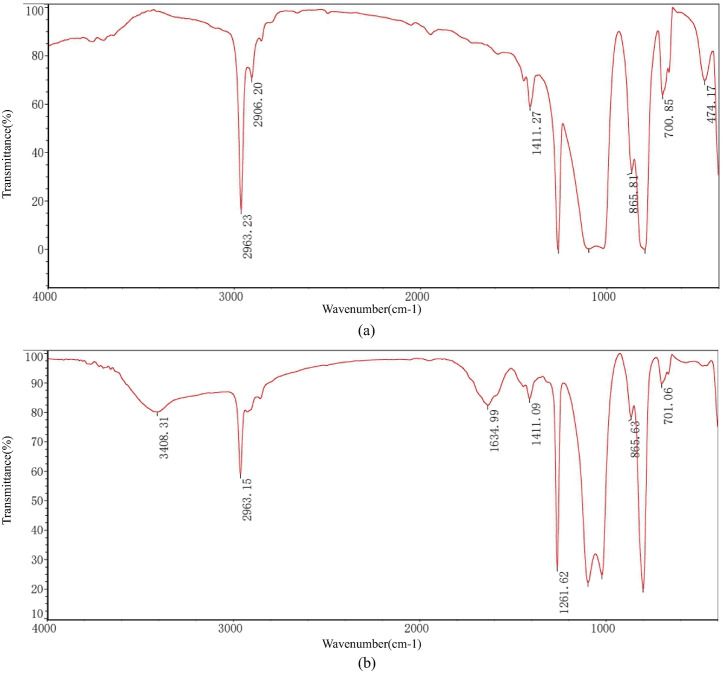
FT-IR spectra ((a) Well W-8, (b) Well W-9).

FT-IR spectroscopy from W-8 and W-9 exhibited elevated organic content. As illustrated in Fig 5, both samples demonstrated functional group characteristics of alkyl and amino groups: aliphatic C-H stretching vibrations were detected within the range of 2963–2906 cm⁻¹, amide bands were observed at 1634 and 1411 cm⁻¹, and C-N/C-O skeletal vibrations appeared at 1260 cm⁻¹. These spectral features collectively confirmed that polyacrylamide constituted the dominant organic component, primarily derived from polymer additives in the pre-injection working fluids.

**Fig 6:**
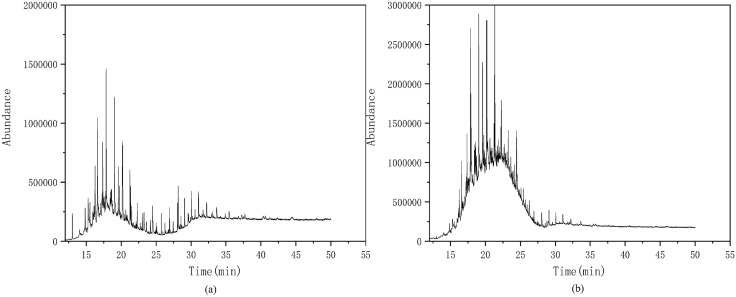
GC-MS test results ((a) Well W-8, (b) Well W-9).

Further, GC-MS analysis was conducted to identify the predominant organic compounds in the plugging samples from representative gas wells W-8 and W-9. As illustrated in Fig 6a, Well W-8 exhibited a notably high concentration of alkane, with long-chain alkanes accounting for 95.2% and lipids accounting for the remaining 4.8%. Fig 6b shows the organic composition of the plugging sample from W-9. Although the alkane content in Well W-9 was relatively lower, long-chain alkanes remained the dominant fraction at 66.9%, while the remaining components consisted of amines and alkynes. GC-MS results aligned well with the results obtained from FT-IR spectroscopy, suggesting that the organic components likely originated from incompletely degraded fracturing fluid gels and drilling fluid that entered the wellbore.

Gas well plugging in the study area exhibited overall similarity with regional variations across different blocks.As shown in Table 3, in Block W2, inorganic components averaged 73.3%, dominated by iron oxides with minor FeS; organic components constituted only 11.9%. Block W4 showed a lower inorganic content of 54.3%, while the organic component increased to 23.8%. Across these blocks, iron-bearing compounds served as main components of the plugging materials, accompanied by BaSO₄, CaCO₃ and trace TiO₂; alongside organic phases dominated by long-chain alkanes.

**Table 3:** Plugging Characteristics Analysis Of Different Blocks.

Block	Well Number	Inorganic component content/%	Organic component content/%	Main characteristic
W2	W-1, W-2, W-3,W-7, W-8	73.342	11.854	Commonly contain iron oxides (e.g., Fe₂O₃, Fe₃O₄) and minor FeS, with organic components primarily composed of long-chain alkanes.
W4	W-4, W-5, W-6,W-9, W-10	54.314	23.766	

The identification of distinct plugging types provides a critical diagnostic basis for field applications. Instead of applying generic treatments, field engineers can now prioritize targeted removal strategies based on preliminary wellbore symptoms and rapid water chemistry analysis, thereby avoiding ineffective treatments and formation damage.

### 3.2. Plugging mechanism

The formation of well plugging is related to downhole corrosion and ionic chemical reactions. The iron-bearing compounds are primarily corrosion products of downhole tubular strings. Carbonate Salt scales form through ionic reactions under fluctuations in temperature and pressure. Furthermore, materials such as barium sulfate and polymeric gels are associated with complex wellbore materials, such as drilling fluids and fracturing fluids.

**Fig 7:**
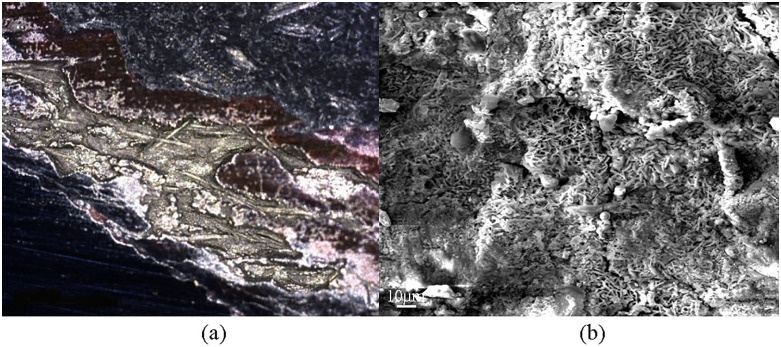
Microscopic images of SRB-corroded tubing pipe ((a) Corroded tubular steel, (b) SEM image of microorganisms on the surface of tubing pipe).

The presence of sulfate-reducing bacteria (SRB) in downhole environments is a primary cause of corrosion-induced plugging [19–20]. Corrosion analysis conducted on Well W-1 tubing string revealed that corrosion occurred on the surface of tubing strings.The corrosion pits were observed to be covered black layer containing elongated objects. As shown in Fig 7, SEM analysis showed that these elongated substances as curved and straight rod-shaped forms, often found in clusters, which highly corresponded to the morphological characteristics of SRB. Bacterial testing of produced water further confirmed abundant SRB, with counts ranging from 60 to 25,000 cells/mL—far exceeding the commonly accepted safety threshold of 25 cells/mL.. SRB are defined as bacteria that can reduce sulfates, sulfites, thiosulfates, and sulfur (S) to hydrogen sulfide (H_2_S), which utilize organics as electron donors to reduce sulfates to sulfides. SRB form colonies on the surface of tubing strings, secreting extracellular polymeric substances that combine with corrosion products to form a black, conductive layer. This layer establishes an electrochemical connection between the metal substrate and the surrounding medium, thereby facilitating electron transfer from the iron substrate to the surface. This process accelerates, cathodic reaction,reslutingn the oxidation of Fe to Fe^2+^ [21]. Simultaneously, the sulfide produced during SRB metabolism reacts with Fe^2+^ to form corrosion products such as FeS. These products accelerate metal corrosion in the weakly acidic formation and under CO_2_ and chloride ion (Cl^-^) conditions, perpetuating a deleterious feedback loop.

In the sulfur-containing environment established by SRB, the subsequent introduction of oxygen may oxidize certain iron sulfides into iron oxides. Additionally, some iron sulfides undergo accelerated oxidation to form iron oxides and deposits.These processes collectively lead to the prevalent presence of iron-bearing compounds in target gas field wells.

Salt scale formation stems from multiple factors affecting the migration of high-salinity formation water. According to the incompatibility theory and thermodynamic equilibrium theory [22,23], the precipitation of solid scale arises from coexistence of incompatible cations (such as Ca^2+^, Ba^2+^) and anions (such as CO_3_^2-^, SO_4_^2-^) in the formation water. When formation water from different zones mixes, solid scale precipitates to maintain system stability. Additionally, during fluid migration to wellhead, changes in temperature, pressure, and oil-gas-water equilibrium can trigger the precipitation of dissolved ions from saturated fluids, leading to salt scale formation. Given the pronounced salt scaling observed in Wells W-8 and W-9, ion analysis of their formation water was conducted, and the results are summarized in Table 4.

**Table 4:** Typical Plugged Gas Well Produced Water Ion Detection Results.

Well No.	K^+^	Na^+^	Ca^2+^	Mg^2+^	Cl^-^	SO_4_^2-^	CO_3_^2-^
W-8	511.5	3008	269	44.2	3421	3.03	103
W-9	30.3	1220	196	26	2376	1.7	65

**Fig 8:**
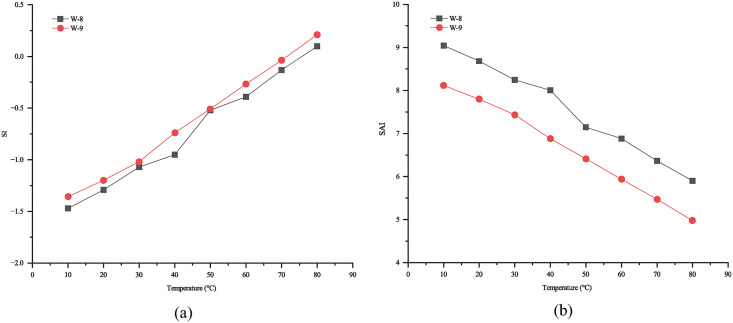
Plugging Prediction ((a) SI method; (b) SAI method).

Combining ion analysis with salt scale sample characterization, CaCO₃ was identified to form via reaction between Ca^2+^ and CO_3_^2-^, precipitating under fluctuations in temperature and pressure.Scaling tendency and severity were predicted using the Davis–Stiff Saturation Index (SI) and Ryznar Stability Index (SAI) methods at varying temperatures in typical gas wells. SAI value between 0 and 6 indicates, indicates a tendency for scale formation. Calculations for the study area predicted CaCO₃ scaling at wellbore temperatures exceeding 80°C. This finding aligns well with the field observations of scale. Results are presented in Fig 8.

As summarized in Table 5, drilling and fracturing operations utilize various fluids- including polymeric drilling muds, oil-based drilling fluids, and slickwater – with polymers serving as the primary constituents. Incomplete degradation of these fluids result in residual polymers whose functional groups, upon exposure to temperature and pressure variations or shear forces, enhance intermolecular interactions, triggering crosslinking and aggregation. This process generates viscoelastic micelles or gels that adhere to the tubing surface. Continued accumulation of these organic deposits ultimately leads to plugging rich in long-chain alkanes, lipids, and amines. Concurrently, barite in drilling fluids [24] precipitates due to solubility changes induced by temperature-pressure fluctuations, further contributing to the progressive well plugging over time.

**Table 5:** Compositional Analysis of Working Fluids and Associated Plugging Components.

Working Fluid	Fluid Type	Main Components	Corresponding Plugging Components
Drilling Fluid	Polymer Drilling Fluid	Polyacrylamide	Degraded into long-chain alkanes (C -C),Oxygen-containing organics (C – O, O – H) and Amines (C – N)
	Polysulfonated Drilling Fluid	Sulfonated Resin, Polyamine Inhibitor, Barite	Oxygen-containing organics (C – O),Amines (C – N), BaSO_4_
	Oil-based Drilling Fluid	Base Oil, Emulsifier	Hydrocarbons (C-H), Oxygen-containing organics (C – O)
Fracturing Fluid	Slickwater	Polyacrylamide	Degraded into long-chain alkanes (C -C),Oxygen-containing organics (C – O, O – H) and Amines (C – N)
		Surfactant	Hydrocarbons (C-H)
		Proppant	SiO_2_

Downhole plugging in the target gas field arises from a self-reinforcing cycle of iron-based corrosion, inorganic scaling, and organic deposition. Sulfate-reducing bacteria initiate anaerobic corrosion, producing H₂S that reacts with iron to form sulfides, which are subsequently oxidized upon oxygen intrusion into iron oxides. Simultaneously, temperature and pressure fluctuations cause incompatible ions in high-salinity formation water to precipitate as carbonate and sulfate scales. Residual polymers from drilling and fracturing operations crosslink under thermomechanical stress. These polymers combine with corrosion products and inorganic scales to form “corrosion product-scale-organic matter” composite plugging bodies. The combined effect of these processes progressively narrows flow channels and leads to persistent wellbore plugging.

### 3.3 Plugging removal agent optimization

Targeted removal agents were developed to address the main types of plugging detected above. In this study, testing samples from Well W-1 were used to optimize iron oxide plugging removal agent, while those for salt scale plugging removal agents optimization tests were sourced from W-8. Dissolution experiments for iron sulfide (FeS) were conducted using actual plugging samples from Well W-10, which were confirmed to contain relatively higher FeS.

**Fig 9:**
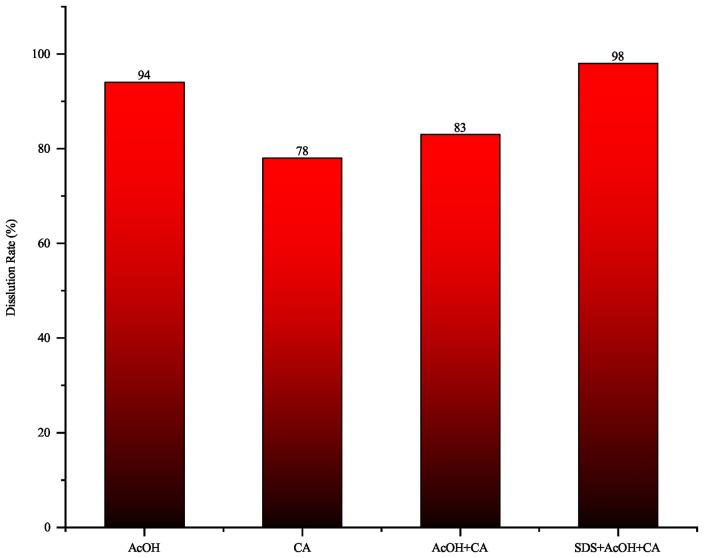
Dissolution rate of four different primary agents on iron oxides.

**Fig 10:**
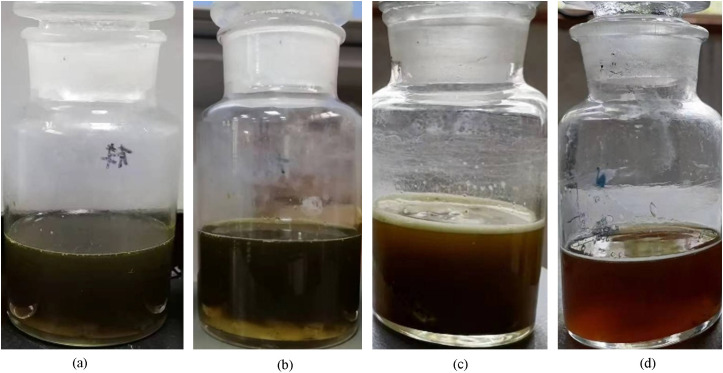
Dissolution performance of four different primary agents on iron oxides ((a) Acetic Acid; (b) Citric Acid; (c) Acetic Acid+Citric Acid; (d) Organic Solvent+Organic Compound Acid).

For plugging problems dominated by iron oxides, dissolution experiments were conducted using a composite approach combining acidification and chelation. Under the experimental conditions, the dissolution perormance is presented in Fig 9. Among the tested systems, an organic solvent combined with an organic composite acid plugging plugging removal agent demonstrated the most effective dissolution, achieving a rate of 98%. This formulation integrates the strong chelating capacity of citric acid with the penetrability of acetic acid,while the organic solvent enhances solubility and dispersibility. The synergy of these three components significantly improves the efficiency of removing iron oxide plugging. The dissolution performance of four different types of iron oxides-based plugging removal agent is shown in Fig 10.

**Fig 11:**
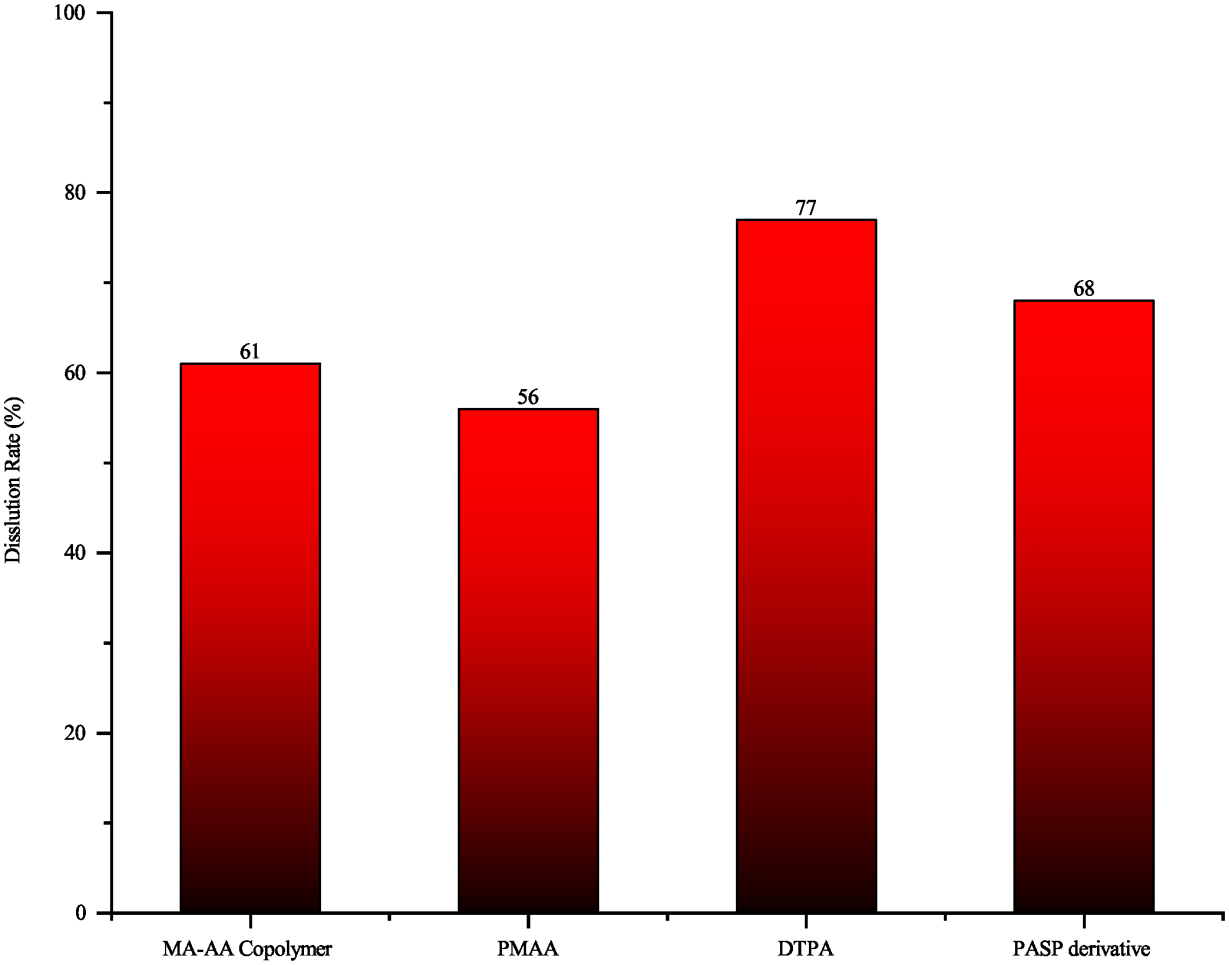
Dissolution rate of four different primary agents on salt scale.

**Fig 12:**
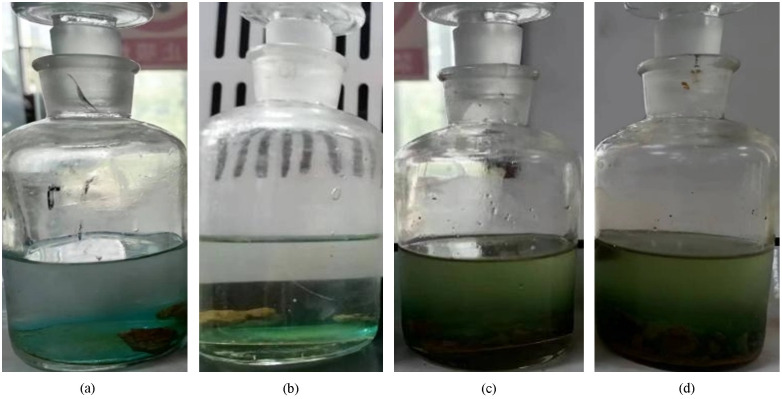
Dissolution performance of four different primary agents on salt scale((a) MA-AA Copolymer; (b) PMAA; (c) DTPA; (d) PASP derivative).

For plugging predominantly composed of salt scale, dissolution experiments were conducted using a formulated blend of chelating agents, leveraging the combined effects of dispersibility and complexation [25,26]. As shown in Fig 11, DTPA as the primary agent exhibited the highest solubility achieving a maximum dissolution rate of 77%. The superior dissolution efficiency of DTPA [27] towards calcium sulfate and calcium carbonate is attributed to its multiple carboxyl and amino groups, which enhance complexation with metal ions and conversion of insoluble salts into soluble complexes. While MA-AA copolymer and PMAA inhibit CaCO₃ deposition via particle dispersion and weak chelation, but show limited effectiveness against BaSO₄. The dissolution performance of four evaluated salt-scale removal agents is shown in Fig 12.

**Fig 13:**
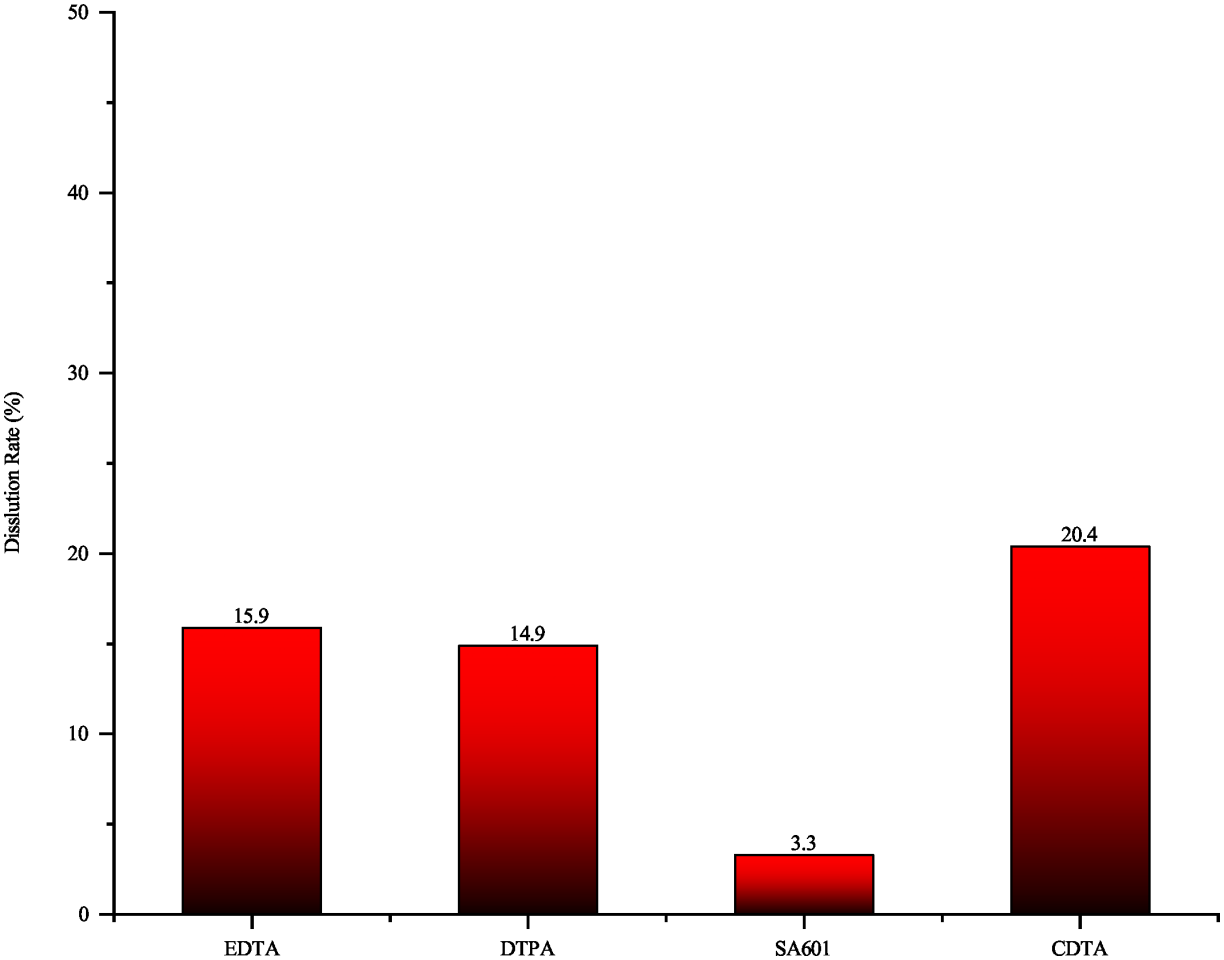
Dissolution rate of four different primary agents on FeS.

**Fig 14:**
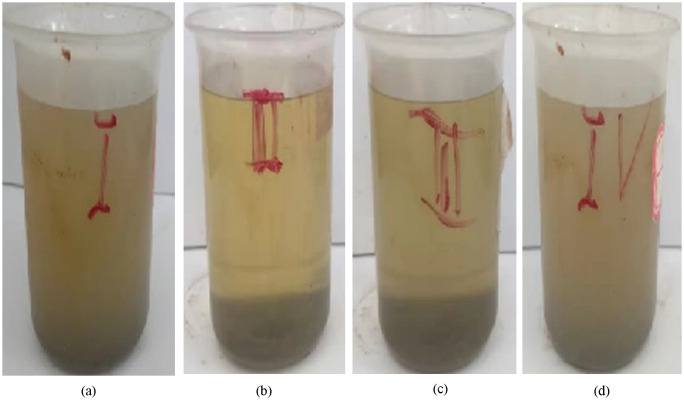
Dissolution performance of four different primary agents on FeS((a) EDTA; (b)DTPA; (c) SA601; (d) CDTA).

**Fig 15:**
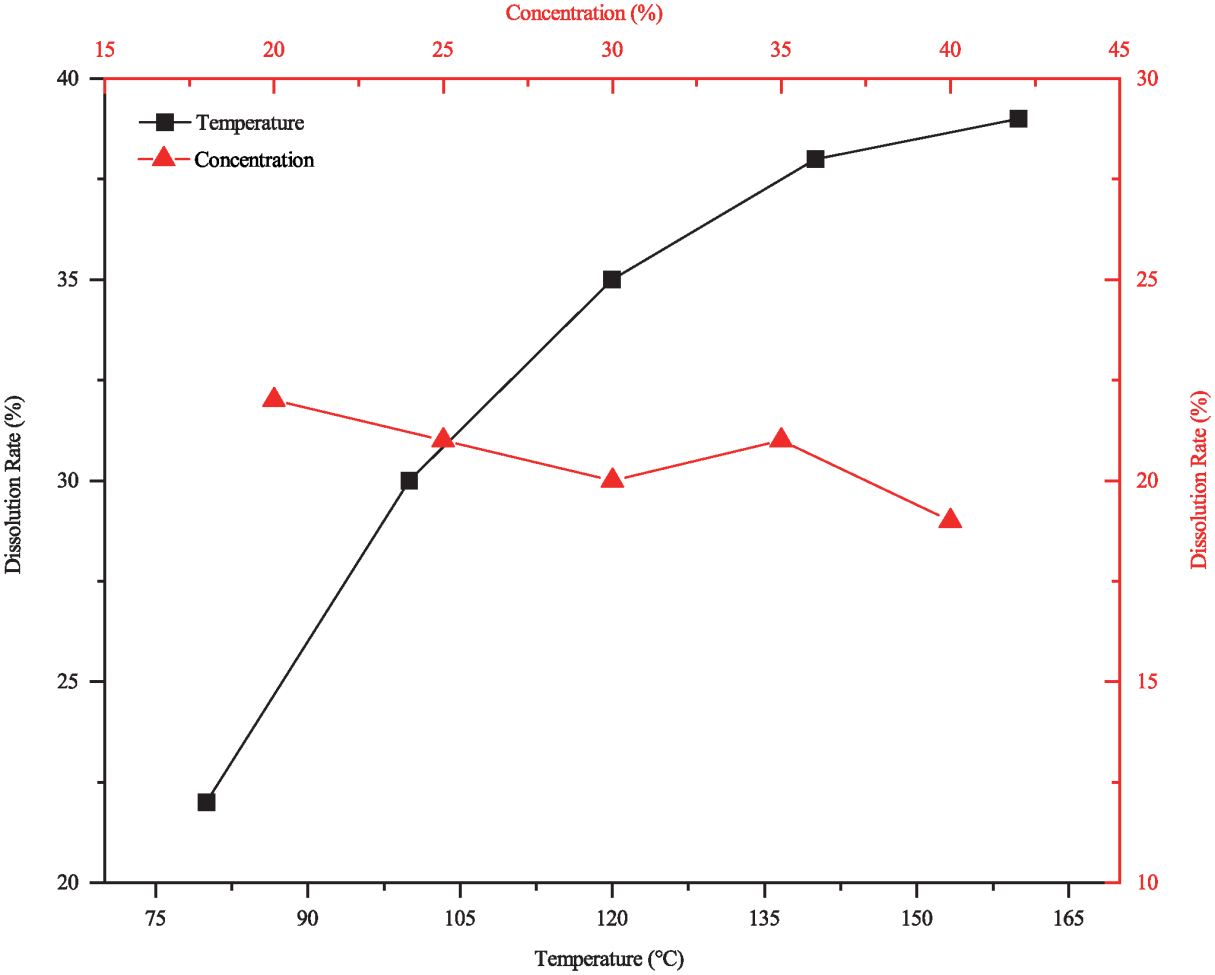
Variation of CDTA Dissolution Rate on FeS with Temperature and Concentration.

The collected plugging samples were accompanied by a small amount of FeS, which can be primarily removed through a combination of penetration and chelation. To select targeted agent, different chelating agents were tested. As shown in Fig 13, CDTA exhibited the best solubility for samples contaning FeS, with a maximum dissolution rate of only 20.4% at experimental temperature. CDTA, a polyamino carboxylic acid chelating agent with more ligand positions [28,29], facilitates electrons transfer to metal atoms, resulting in a relatively high corrosion rate. However, the presence of TiO_2_, a highly stable oxide insoluble in most chelating agents, constituted a substantial fraction of the sample that was unaffected by the treatment. The dissolution performance of four tested removal agents is shown in Fig 14. Increasing CDTA concentration had limited effect on dissolution efficiency, while elevated temperature significantly improved performance. As shown in Fig 15, at a concentration of 20 wt% and a temperature of 160°C, the dissolution rate reached 39.3%.

Corrosion rates of three selected plugging removal agents on N80 steel coupons were tested at temperatures ranging from 25 to 120°C, based on the well temperatures in the target shale gas field.As shown in Table 6, the plugging removal agent for iron-oxide plugging exhibited relatively high corrosion rates, reaching 18.5 g/(m^2^·h) at 120°C. At temperatures below 100°C, the corrosion rates were relatively low, meeting the standard requirements for corrosion rates on N80 steel. Although corrosion rates increased above 120 °C, the surface of the steel coupons remained smooth, flat, and shiny, still satisfying field application requirements.

**Table 6:** Iron Oxide-Based Plugging Removal Agents Corrosion Test Results.

Temperature,°C	25	40	60	80	120
Corrosion Rate,g/(m^2^·h)	0.41	0.50	0.66	0.82	18.5

The corrosion rate of the chelating agent-based plugging removal system was lower than that of iron-based plugging removal agent. As shown in Table 7,under 120°C, the corrosion rates of iron sulfide-based and salt scale-based plugging removal agents on N80 steel coupons were 1.15 g/(m^2^·h) and 6.71 g/(m^2^·h), respectively. The corroded surfaces remained smooth and uniform, with no evidence of pitting corrosion.

**Table 7:** Chelating Plugging Removal Agents Corrosion Test Results.

Type of Plugging Removal Agent	Temperature,°C	Corrosion Rate, g/(m2·h)
Iron Sulfide-Based	80	0.69
	120	1.15
Salt Scale-Based	80	3.83
	120	6.71

Targeted chemical removal strategies have been developed for field implementation. The optimized dissolution agents—such as citric acid, acetic acid, DTPA, or CDTA—are selected according to the predominant plugging type and are combined with iron stabilizers, surfactants, clay stabilizer and other additives to create a composite remover. This formulation exploits synergistic acidification and chelation mechanisms to efficiently dissolve deposits while mitigating corrosion and preventing secondary precipitation.

Building upon the laboratory-based analysis of plugging composition and mechanism, along with the optimized design of removal agents, a field-implementable chemical flushing protocol has been established for plugged wells with preserved fluid pathways. Utilizing pumping equipment such as pump truck, the tailored agent is injected into the wellbore following a systematic procedure:(1) initial injectiing through the tubing to chemically remove plugging near the wellbore, (2) extending treatment via casing injection,(3)shutting in the well for about 24h to allow sufficient reaction,(4) controlled flowback to recover dissolved solids. This approach effectively translates laboratory findings into a reliable field solution that enhances production recovery while minimizing operational impact, thereby bridging the gap between mechanistic understanding and practical well intervention.

## 4. Conclusions

This study comprehensively investigated the plugging mechanisms and chemical treatment strategies in shale gas wells from the Weiyuan field through integrated experimental and analytical approaches. The main findings are as follows:

Plugging materials were predominantly inorganic, consisting mainly of iron-containing compounds, calcium carbonate, and barium sulfate. Organic components—present in minor proportions—included residual gelled fracturing fluid derivatives such as long-chain alkanes and polyacrylamide. Corrosion-induced iron-based deposits originated from sulfate-reducing bacterial activity, yielding Fe^2+^/Fe^3+^ ions that precipitated as oxides/sulfides. Inorganic scaling resulted from supersaturation and crystallization of incompatible ions (e.g., Ca^2+^, Ba^2+^, CO₃^2^⁻, SO₄^2^⁻) under changing pressure-temperature conditions, while organic residues were linked to incomplete degradation of injected fluids.

Targeted removal agents were developed based on compositional insights: an organic composite acid–organic solvent system achieved 98% dissolution efficiency for iron oxides; a CDTA-based chelator was optimal for iron sulfides; and DTPA demonstrated the highest efficacy against barium sulfate/calcium carbonate scales, reaching a dissolution rate of 77%. Corrosion tests confirmed that all systems exhibited acceptable corrosion rates without pitting, meeting field application standards under downhole temperature conditions.

These findings provide a scientific basis for the design of effective and reliable chemical interventions tailored to specific plugging types in shale gas production systems.

## Supporting information

S1 Data Supporting Data. (XLSX)
